# The Agreement Rate between Radiographic Interpretation and Histopathologic Diagnosis of Jaw Lesions

**DOI:** 10.1155/2019/4056359

**Published:** 2019-03-04

**Authors:** Soulafa Almazrooa, Nada O. Binmadi, Hanadi M. Khalifa, Fatima M. Jadu, Ahmed M. Jan, Dalia E. Meisha

**Affiliations:** Faculty of Dentistry, King Abdulaziz University, Jeddah, Saudi Arabia

## Abstract

**Background:**

To determine the agreement rate between histopathologic diagnosis and radiographic interpretation of jaw lesions.

**Methods:**

Cases with jaw pathologies that have diagnostically adequate histopathologic samples and radiographic examinations were reviewed retrospectively. Two board-certified oral and maxillofacial pathologists (OMFP) independently determined the histopathologic diagnosis, while two board-certified oral and maxillofacial radiologists (OMFR) determined the radiographic interpretations independently. Then the histopathologic diagnosis and the radiographic interpretation were compared for agreement.

**Results:**

A total of 104 cases (53% females) were included with a mean age of 31 years. The agreement rate between histopathologic diagnosis and radiographic interpretation was 49%. OMFP required OMFR consultations to reach a diagnosis in 16% of cases. The most commonly encountered lesions were by far odontogenic cysts of inflammatory origin and the agreement for this disease category was 49.1%. However, agreement rates were highest for the disease category of tumors (62.5%).

**Conclusion:**

The agreement rate between OMFP and OMFR was higher for tumors than cysts. Agreement rates between OMFP and OMFR improved with efficient consultation between the two disciplines.

## 1. Introduction

The oral and maxillofacial area is a complex region with many tissues and structures and is thus the site of much diverse pathology. Some of these pathologies affect the soft tissues, while others affect the osseous tissues and others affect both tissues. The clinical and radiographic findings play a significant role in diagnosis but histopathology is usually the major determinant of the diagnosis.

Biopsy is the gold standard for the diagnosis of many jaw pathologies [1]. The diagnosis assigned by a pathologist at the end of a biopsy report is often the basis on which many crucial decisions are based in regard to patient management and prognosis. Yet, the accuracy of a pathologists' ability to reach the correct diagnoses is an inadequately studied area. In the literature, the highest misdiagnosis rate was related to non-odontogenic tumors of the oral cavity, accounting for approximately 11.5%, followed by malignant tumors, accounting for approximately 9% [2].

The overall concordance between clinical findings and histopathologic diagnosis ranges between 50 and 99% [2–4]. However, there is no data on the concordance between radiographic interpretation and histopathologic diagnosis of jaw lesions. Therefore, in the current study, we investigated the agreement rate between radiographic interpretation and histopathologic diagnosis of jaw lesions.

## 2. Materials and Methods

In this retrospective review, the records of all patients referred to a university-based oral and maxillofacial pathology service over a three-year period were reviewed. Inclusion criteria included the availability of diagnostically adequate histopathologic samples and diagnostically adequate imaging with reports of jaw lesions based on the 2017 World Health Organization (WHO) classification of odontogenic and maxillofacial lesions [5]. This descriptive study took place at the Faculty of Dentistry of King Abdulaziz University, Jeddah, Saudi Arabia. Data collection commenced after ethical approval from the research ethics board (Number 009-15) was obtained. The guidelines of the Helsinki Declaration were followed diligently. Each patient was assigned a unique number that linked the clinical, radiographic, and histopathologic information. However, this information was available only to the principal investigator so that no connection can be made to the patient.

Two board-certified oral and maxillofacial pathologists (OMFP) reviewed the histopathology samples and their diagnosis was based on the 2017 World Health Organization (WHO) classification of odontogenic and maxillofacial bone lesions [5]. Two board-certified oral radiologists (OMFR) reviewed and interpreted the radiographic images. Brief clinical information was available to both disciplines as age, gender, location, and pertinent medical history, if any. The histopathology requisition form did not consistently include reference to the availability of imaging for each case.

All available images were reviewed including periapical and bitewing radiographs, panoramic radiographs, cone beam computed tomography (CT), multidetector CT, and magnetic resonance imaging (MRI) examinations. Disagreements among the disciplines were resolved by consensus.

Cases were allocated into disease categories consistent with the 2017 World Health Organization (WHO) classification of odontogenic and maxillofacial lesions [5]. Then, the histopathologic diagnosis was compared to the radiographic interpretation and each case was given one of three codes based on the agreement between the two disciplines: (0) no agreement, (1) agreement, and (2) if communication was needed between the two disciplines to reach a diagnosis. Statistics were done using the Statistical Package for Social Sciences (SPSS 22, Windows, SPSS Inc., Chicago, USA). Percent agreement was calculated.

## 3. Results

The initial sample included 311 cases. Of these, only 128 cases (41%) had a diagnostically adequate histopathologic sample. Furthermore, eighty-one percent of cases of the 128 cases had diagnostically adequate images. One hundred and four cases were included in this study.

Table 1 exhibits the descriptive statistics for the cases included in the study sample. The mean age was 31 years with a wide range of age from 4 years to 76 years. There was almost equal representation in terms of gender and location of the pathology (maxilla versus mandible). In terms of the disease categories, the most prevalent category was odontogenic cysts of inflammatory origin (55%), while fibroosseous lesions (FOL) were the least prevalent (2.9%). Only 18% of cases had some form of advanced imaging defined as any three-dimensional imaging such as computed tomography, cone beam computed tomography, or magnetic resonance imaging.

**Figure 1:**
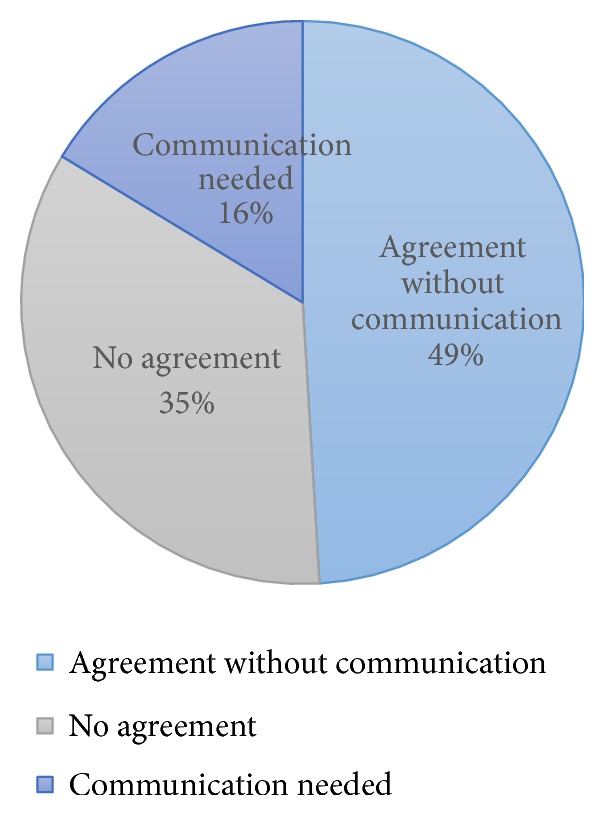
Agreement and need of communication to reach a diagnosis between oral and maxillofacial pathologist (OMFP) and oral and maxillofacial radiologist (OMFR).

In sixteen percent of cases, OMFP required OMFR consultation to reach a diagnosis. Agreement between OMFP and OMFR was demonstrated in almost half of the cases (49%), whereas no agreement was reached in 35% of cases (Figure 1).

**Figure 2:**
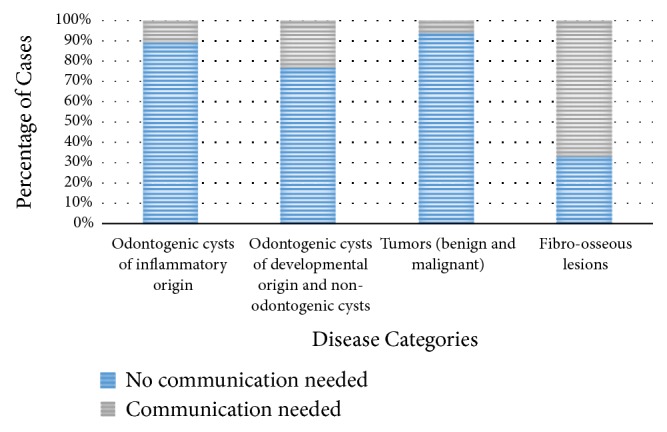
Comparison of percentage of cases that did and did not require communication between oral and maxillofacial pathologist (OMFP) and oral and maxillofacial radiologist (OMFR) in relation to disease categories.

The need for communication between OMFP and OMFR varied according to the disease category. Communication was not needed for 93.8% of cases under the tumors category and 89.1% of cases under the odontogenic cysts of inflammatory origin category (Figure 2). The odds of needing communication between OMFP and OMFR were about 2.5 times higher for odontogenic cysts of developmental origin and non-odontogenic cysts compared to odontogenic cysts of inflammatory origin but did not reach statistical significance (odds ratio (OR): 2.5, 95% CI of OR: 0.7-8.2). While the odds of needing communication were half for tumor cases compared to cases with odontogenic cysts of inflammatory origin (OR: 0.5, 95% CI of OR: 0.06- 4.9). The need of communication between OMFP and OMFR was three times more with cases having advanced imaging compared to cases with conventional imaging only (OR: 3.2, 95% CI of OR: 1-10.4).

**Figure 3:**
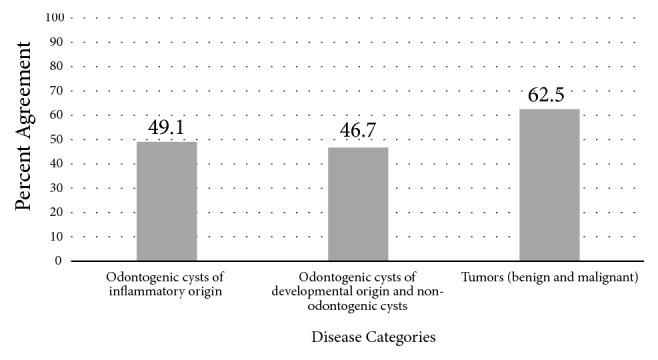
Percent agreement between histopathologic diagnosis and radiographic interpretation based on disease categories.

The percent agreement between OMFP and OMFR in this sample varied according to the disease category and was highest for the tumors category (62.5%). This was followed by odontogenic cysts of inflammatory origin (49.1%) and odontogenic cysts of developmental origin as well as non-odontogenic cysts (46.7%). For the FOLs disease category, there was no agreement at all between OMFR and OMFR. These results are demonstrated in Figure 3. The odds of agreement between OMFP and OMFR were about twice times higher for tumors compared to odontogenic cysts of inflammatory origin but did not reach statistical significance (OR= 1.7, 95% CI of OR: 0.6- 5.4). While the odds of agreement were almost comparable for cysts of developmental origin compared to odontogenic cysts of inflammatory origin cases (OR= 0.9, 95% CI of OR: 0.4-2.2).

## 4. Discussion

Reaching an accurate diagnosis is a fundamental step in the process of patient management and treatment planning. The process of reaching a correct diagnosis is not an easy one and proceeds through several stages that requires input from numerous outlets including clinical findings, imaging findings, and histopathology findings. Therefore, it is essential that these data be collected in a thorough manner to ensure completeness. It is also crucial that these data be correlated to ensure comprehensiveness.

Cohen's kappa is the most commonly used measure to assess inter-rater reliability [6]; however Kappa was incalculable in this study because the histopathologic diagnosis is the gold standard and therefore was constant. This is a recognized limitation of Kappa [7]. Percent agreement was calculated instead and was found to be 49% between radiographic interpretation and histopathologic diagnosis, which is lower than other published studies. A study by Sarabadani et al. in 2013 found the concordance between radiology and histopathology for central jaw lesions to be 71.4% [8], whereas the concordance between clinical impression and histopathology diagnosis was 80.4% [8]. Several studies have investigated the concordance between clinical and histopathology without any regard for imaging data. This may be due to the few number of cases with available imaging.

The most commonly encountered disease category was odontogenic cysts of inflammatory origin. This disease category accounted for 53% of cases included in this review; however, the agreement rate for this entity was only 49%. This is due to the fact that differentiating periapical granulomas from cysts is challenging from an imaging perspective. From a practical perspective, this differentiation may not be necessary since both conditions are usually treated similarly. Agreement rates were highest for tumors perhaps because the features of these lesions are undebatable or perhaps because the reviewers were not required to assign a specific disease label. This measure was taken because from an imaging perspective it is usually easy to recognize malignant features but it is difficult to pinpoint the exact type of malignancy.

The radiographic images that were examined in the current study were not limited to a specific type or number and included any images that were available for each case. These included conventional images as well as advanced imaging, such as cone beam computed tomography (CBCT), conventional computed tomography (CT), and magnetic resonance imaging (MRI). The accuracy of each type of imaging varies, which ultimately influences the accuracy of the interpretation. We predict that the agreement between radiographic interpretation and histopathology diagnosis may have been higher, had all the available images been of the advanced type. Advanced imaging is the standard of care in most cases with pathology because the three-dimensional imaging provides significantly more information regarding the features, behavior, and extent of the lesion, which improves the interpretation process. Future studies should examine the effect of imaging type on the agreement rate with histopathologic diagnosis.

Approximately 16% of the osseous lesions reviewed in this study required communication between OMFP and OMFR before a final histopathologic diagnosis was assigned. This was especially true for FOL as 67% required communication with the radiologist. FOL lesions such as fibrous dysplasia for example are known to be challenging in terms of histopathologic diagnosis, their features can be confused with those of other forms of fibro-osseous lesions and with osteomyelitis and even well differentiated osteogenic sarcoma [9–11]. Three vastly different conditions with extremely different management approaches. For these cases, imaging plays a significant role and communication between the OMFP and OMFR becomes of paramount importance.

Gephardt at al. reported that missing information such as the anatomic location of the lesion can result in revision or even changing of a histopathologic diagnostic decision [12]. Furthermore, other studies have demonstrated that amended histopathology reports after consultation with the clinician or radiologist lead to major changes in management in 3.8% of cases and minor changes in 2.9% of cases [13]. Therefore, it is essential for OMFP to communicate efficiently with all specialists involved in the patients' care, including clinicians, surgeons, and radiologists, to obtain all necessary information for an accurate diagnosis, especially if the necessary information is missing from the requisition form [12, 14, 15]. The simple act of communication has the potential to significantly reduce the chances of misdiagnosis and to ultimately improve patient outcome. Therefore, we strongly advocate all methods of communication, whether verbal or non-verbal, electronic or otherwise as long as they are eventually documented. In addition, we strongly advocate that each healthcare institution has specific policies on communication between specialists.

Limitations of this study include that the clinical differential diagnosis details included in the pathology requisition form were brief and inconsistent. This prevented the possibility of investigating the concordance between the clinical diagnosis and the radiographic impression with histopathology diagnosis. Although the role of clinical findings was not examined in this study, it should not be undermined or overlooked. Another limitation is having a relatively small proportion of cases identified as tumors (15.4%). This limited the possibility of assessing the differences in agreement between benign and malignant tumors, if any. In addition, the generalizability of the agreement rate for the FOL disease category was limited because only 3% of cases were diagnosed as FOL.

## 5. Conclusion

Agreement rates between OMFP and OMFR were higher for tumors when compared to cysts. Also, agreement rates between OMFP and OMFR improved with efficient consultation between the two disciplines. Future studies should aim to investigate the agreement among clinical, radiographic, and histopathologic findings.

## Figures and Tables

**Table 1:** Descriptive statistics for the cases included in the study (N=104).

*Study Variable*	*Descriptive Statistics*
	*Mean * + * SD (range)*
*Age* (years)	30.7+ 16.2 (4-76)

	*% (N)*

*Gender* (female)	53.8% (56)

*Location* (mandible)	53.8% (56)

*Disease category:*	

(i) Odontogenic cysts of inflammatory origin	52.9% (55)

(ii) Odontogenic developmental cysts and non-odontogenic cysts	28.8% (30)

(iii) Tumors (benign and malignant)	15.4% (16)

(iv) Fibro-osseous lesions	2.9% (3)

*Advanced imaging*	18.3% (19)

SD: standard deviation; advanced imaging: any three-dimensional imaging such as computed tomography, cone beam computed tomography, or magnetic resonance imaging.

## Data Availability

The data used to support the findings of this study are included within the supplementary information files.

## Supplementary Materials

Supplementary Materials The supplemental material is the data on which the findings of the study are based. I submitted it as supplemental material as per the author instruction.
